# Multiple potential roles of thymosin β4 in the growth and development of hair follicles

**DOI:** 10.1111/jcmm.16241

**Published:** 2021-01-03

**Authors:** Bai Dai, Ri‐Na Sha, Jian‐Long Yuan, Dong‐Jun Liu

**Affiliations:** ^1^ Key Laboratory of Reproductive Regulation and Breeding of Grassland Livestock School of Life Sciences Inner Mongolia University Hohhot China; ^2^ Reproductive Medicine Center The Affiliated Hospital of Inner Mongolia Medical University Hohhot China; ^3^ Pathology department The Affiliated Hospital of Inner Mongolia Medical University Hohhot China; ^4^ Clinical laboratory The Affiliated Hospital of Inner Mongolia Medical University Hohhot China

**Keywords:** development, growth, hair follicle, thymosin β4

## Abstract

The hair follicle (HF) is an important mini‐organ of the skin, composed of many types of cells. Dermal papilla cells are important signalling components that guide the proliferation, upward migration and differentiation of HF stem cell progenitor cells to form other types of HF cells. Thymosin β4 (Tβ4), a major actin‐sequestering protein, is involved in various cellular responses and has recently been shown to play key roles in HF growth and development. Endogenous Tβ4 can activate the mouse HF cycle transition and affect HF growth and development by promoting the migration and differentiation of HF stem cells and their progeny. In addition, exogenous Tβ4 increases the rate of hair growth in mice and promotes cashmere production by increasing the number of secondary HFs (hair follicles) in cashmere goats. However, the molecular mechanisms through which Tβ4 promotes HF growth and development have rarely been reported. Herein, we review the functions and mechanisms of Tβ4 in HF growth and development and describe the endogenous and exogenous actions of Tβ4 in HFs to provide insights into the roles of Tβ4 in HF growth and development.

## 1 INTRODUCTION

**FIGURE 1:**
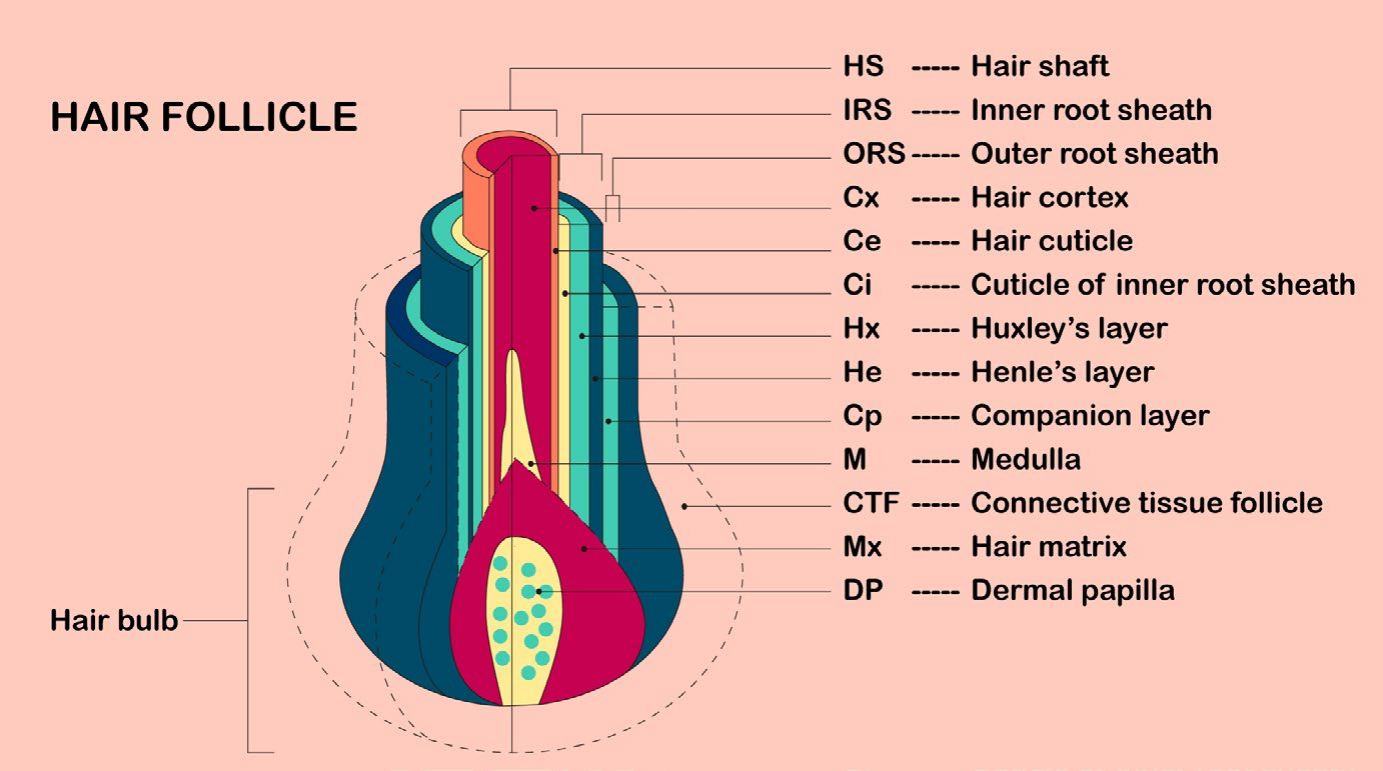
Schematic representation and terminology of the structure of the hair follicle. The hair shaft (HS) consists of the medulla (M), hair cortex (Cx) and hair cuticle (Ce). The inner root sheath (IRS) consists of the cuticle of the inner root sheath (Ci), Huxley's layer (Hx) and Henle's layer (He). The companion layer (Cp) is occasionally referred to as a member of the outer root sheath (ORS), which includes the hair matrix (Mx) and dermal papilla (DP)

The hair follicle (HF) is an important mini‐organ of the skin, consisting of nine distinctive epidermal layers, that is hair matrix, medulla, hair cortex, hair cuticle, cuticle of the inner root sheath, Huxley's layer, Henle's layer, companion layer and outer root sheath, arranged concentrically from core to periphery, as well as two dermal tissues, that is dermal papilla (DP) and connective tissue follicle^1^ (Figure 1). These structures are predominantly comprised of five types of cells: concentric ringed epithelial cells that form the hair shaft and inner root sheath,^2^, ^3^ HF stem cells and their progenitor cells in the bulge region,^4^, ^5^, ^6^ matrix cells at the bulbar base^7^ and mesenchymal cells surrounded by the matrix (DP cells [DPCs]),^5^ as well as melanocytes, which give pigment to the hair, and sebocytes, which make up the mature sebaceous gland.^8^ HF formation occurs throughout the development of embryonic skin and depends on stepwise signalling between the epithelial epidermis and mesenchymal dermis.^7^ DPCs are important signalling components that guide the proliferation, upward migration and differentiation of HF stem cell progenitor cells to form other types of HF cells.^9^, ^10^ Thus, these cells are tightly controlled during the growth and development of HFs, both temporally and spatially.^11^ Moreover, the growth and development of HFs occur via a cycle of three continuous stages, that is hair shaft formation and rapid growth (anagen phase), apoptosis‐driven hair shaft degradation (catagen phase), and hair disappearance and relative quiescence (telogen phase).^12^, ^13^

Previous studies have shown that HF and keratinocyte components may promote hair growth when the amount of DPCs reaches a certain threshold.^14^, ^15^ Therefore, the enhancement of the proliferation ability of DPCs is an effective means to promote hair growth. Currently, three main types of strategies are available to promote hair growth through DPCs. First, the proliferation of DPCs can be promoted by treatment with certain medications. Kang et al^16^ found that mackerel‐derived fermented fish oil promotes the proliferation of DPCs by activating Wnt/β‐catenin signalling for hair growth. Second, the proliferation of DPCs is promoted by the overexpression or inhibition of endogenous genes. Wu et al^17^ found that Wnt10b promotes DPC proliferation in Rex rabbits. In contrast, Yu et al^18^ demonstrated that mitotic arrest‐deficient protein 2B plays negative roles in T‐cell factor 4–induced DPC proliferation in human beings. Finally, hair growth is promoted by increasing the secretion of extracellular vesicles (EVs) by DPCs.^19^ Kwack et al^20^ reported that EVs derived from DPCs promote hair growth and hair regeneration by regulating the activity of follicular dermal and epidermal cells. Additionally, Yan et al^21^ demonstrated that EV microRNAs derived from DPCs mediate the proliferation and differentiation of HF stem cells.

Despite significant advances in molecular technologies, the molecular basis of hair growth promotion through DPCs is still not well understood. Hair growth resembles wound healing in that it requires a highly coordinated interplay among cell proliferation, cell differentiation and cell migration.^22^, ^23^ The transformation of HF telogen and anagen stages depends on the crosstalk of DPCs and HF stem cells to produce the necessary activators.^24^, ^25^ Previous studies have shown that DPCs promote hair growth by secreting Wnt/β‐catenin,^26^ Notch,^27^ bone morphogenetic protein^28^ and sonic hedgehog (Shh)^29^, ^30^ signalling molecules, which communicate with epithelial cells.^24^ In particular, the canonical Wnt/β‐catenin signalling pathway plays critical roles in facilitating HF entry into the anagen stage.^31^ Similarly, during wound healing in mice with cutaneous injury, Shh levels rise, activating the hedgehog pathway and promoting HF regeneration. Therefore, the overexpression of Shh on the epidermis can lead to extensive HF regeneration in wounds, suggesting that the activation of the Shh signal in Wnt‐responsive cells can promote wound healing.^32^

These events are driven by compartmentalized cell types and their signalling molecules, which regulate de novo hair formation in embryonic skin and new hair growth cycling in adult skin, switching from dormancy to rapid cell division during cycling.^33^ Therefore, the search for key signalling molecules that control these processes has become a major focus of studies of the growth and development of HFs.

Thymosin β4 (Tβ4) is a highly conserved G‐actin–sequestering protein that is involved in various biological processes, such as cell migration, angiogenesis and wound healing. Recently, Tβ4 has been shown to play key roles in HF growth and development. Moreover, endogenous Tβ4 can activate the HF cycle transition in mice and affect HF growth and development by promoting the migration and differentiation of HF stem cells and their progeny.^34^, ^35^ In addition, exogenous Tβ4 increases the rate of hair growth in mice and promotes cashmere production by increasing the number of secondary HFs in cashmere goats.^36^, ^37^, ^38^ However, the molecular mechanisms through which Tβ4 promotes HF growth and development have not been described in detail.

Herein, we review the functions and mechanisms of Tβ4 in HF growth and development and describe the endogenous and exogenous actions of Tβ4 in HFs to provide insights into how Tβ4 mediates HF growth and development.

## 2 THE STRUCTURE AND DISCOVERY OF Tβ4

Thymosins are lymphoid growth factors that were first isolated by Goldstein and White from the thymus of a calf in 1966.^39^, ^40^ Based on the isoelectric point (pI), thymosins can be classified into α (pI < 5.0), β (5.0 < pI <7.0) and γ (pI > 7.0) types.^41^, ^42^ Among these types, thymosin β, characterized by sequestration of globular actin (G‐actin) monomers, is highly conserved at the amino acid sequence level in species ranging from echinoderms to mammals.^43^ Currently, 16 thymosin β members have been reported; among these, Tβ4, thymosin β10 (Tβ10) and thymosin β15 (Tβ15) have been identified and isolated in mammals. Studies have shown that Tβ4, accounting for more than 70% of all thymosins, is found ubiquitously in mammalian body fluids, tissues and cells (except erythrocytes) and can be detected in both the nucleus and cytoplasm.^41^

**FIGURE 2:**
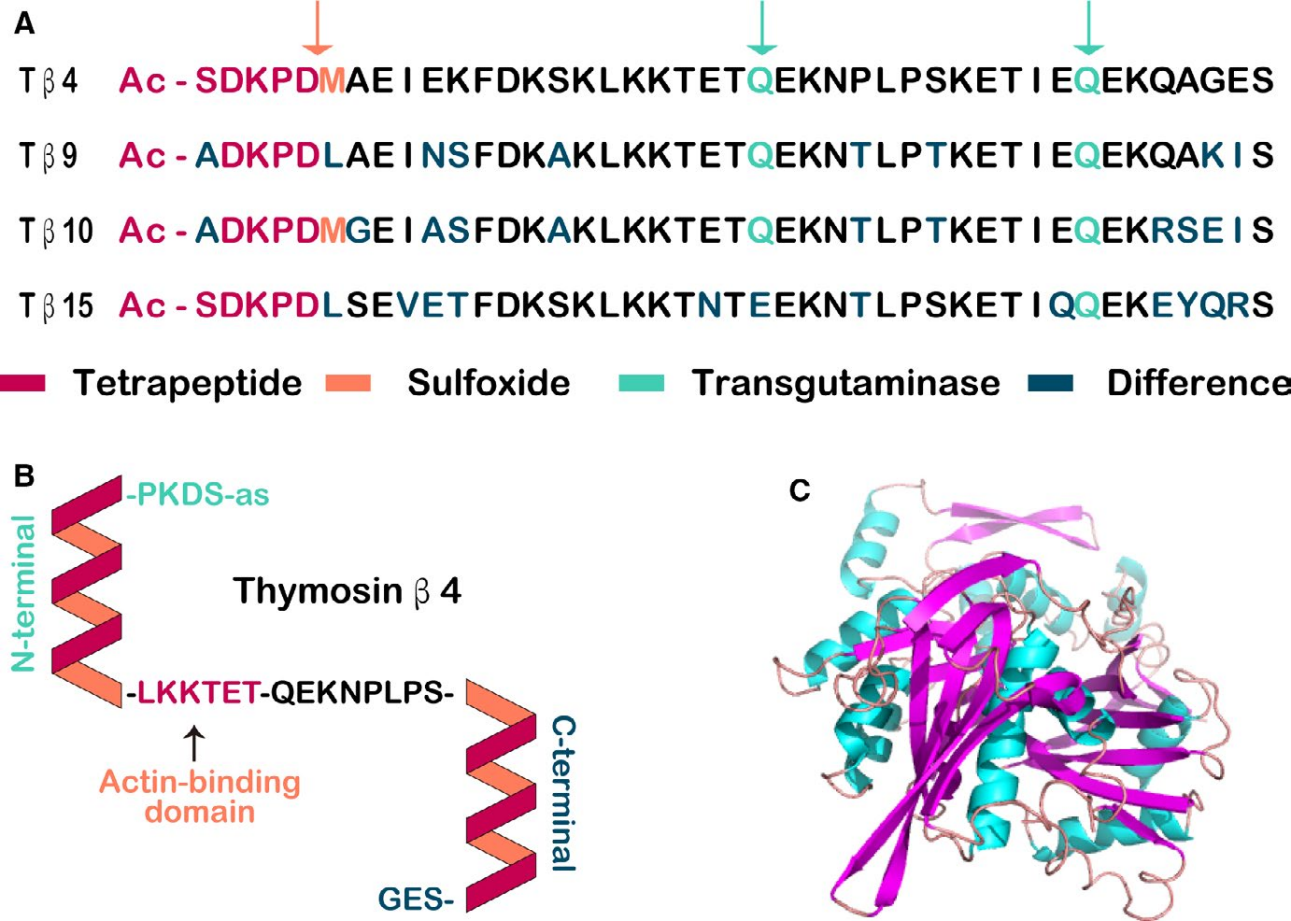
Schematic representation of β‐thymosins. (A) Comparison of β‐thymosins expressed in human beings. Proteolytic cleavage (orange arrow) leads to the generation of an acetylated tetrapeptide. Methionine residues in Tβ4 and Tβ10 can be oxidized to methionine sulfoxide residues (orange letters). Glutamine residues (green labelled with green arrows) are glutaminyl donors in transglutaminase reactions. (B) The secondary structure of Tβ4. Thymosins in aqueous solution are unstructured and flexible. The actin‐binding motif (‐LKKTET‐) is responsible for the initial interaction with G‐actin, which induces the formation of two helices (shown simplified). Residues 1‐5 contact subdomain 3 of G‐actin. The N‐terminal helix of Tβ4 contacts subdomain 1 of G‐actin. The residues between the N‐terminal and C‐terminal helices expand on subdomain 2. The C‐terminal helix contacts subdomain 2 and may close the cleft between subdomains 2 and 4. Tβ4 binds to G‐actin in an extended conformation and thus blocks salt‐induced actin polymerization by steric hindrance. (C) Possible 3D structure of Tβ4. The structure for Tβ4 was visualized using the PyMOL program

Tβ4 is a water‐soluble G‐actin–sequestering protein with a molecular weight of 4.9 kDa and a pI of 5.1 (Figure 2). Tβ4 is composed of 43 amino acid residues, the most abundant of which are glutamate and glycine.^44^, ^45^ Protein conformation analysis has shown that there is a high helical content at positions 4‐15 and 30‐40 of the amino acid sequence of Tβ4. Additionally, internal overlap is observed at positions 18‐30 and 31‐43. N‐acetyl‐seryl‐aspartyl‐lysyl‐proline is the N‐terminal amino acid residue sequence of Tβ4, which can be obtained by direct cleavage of the chemical bond between proline and aspartic acid using prolyl oligopeptidase or Aspn‐like protease.^46^ Nuclear magnetic resonance analysis showed that Tβ4 mainly contains α helices in aqueous solution, exhibiting a loose structure. When the pH value of the aqueous solution is 4.5‐7.5 and the temperature is between 5 and 40°C, the secondary structure of Tβ4 is difficult to identify. In addition to its binding affinity to G‐actin, Tβ4 is able to form complexes with F‐actin.^47^

In recent years, Tβ4 has been extensively studied, mainly owing to its involvement in various biological processes. In 1995, Grant and colleagues reported that Tβ4 is involved in endothelial cell differentiation.^48^ In addition to its ability to induce endothelial cell differentiation, Tβ4 also promotes endothelial cell migration and stimulates microtubule formation and angiogenesis in vitro and in vivo. Moreover, Tβ4 enhances wound healing through various mechanisms, including increased angiogenesis, improved keratinocyte migration, collagen deposition and wound contracture.^49^, ^50^ Furthermore, Tβ4 has anti‐inflammatory and hair growth improvement effects.^35^ Recently, Tβ4 has been shown to play key roles in HF growth and development. Endogenous Tβ4 can activate the HF cycle transition in mice and affects HF growth and development by promoting the migration and differentiation of HF stem cells and their progeny.^34^, ^35^ In addition, exogenous Tβ4 increases the rate of hair growth in mice and promotes cashmere production by increasing the number of secondary HFs in cashmere goats.^36^, ^37^, ^38^ Taken together, these findings demonstrate that Tβ4 is a pleiotropic protein with important roles in the growth and development of HFs via modulation of HF growth and development through its various functions.

## 3 ROLES OF ENDOGENOUS Tβ4 IN HF GROWTH AND DEVELOPMENT

**FIGURE 3:**
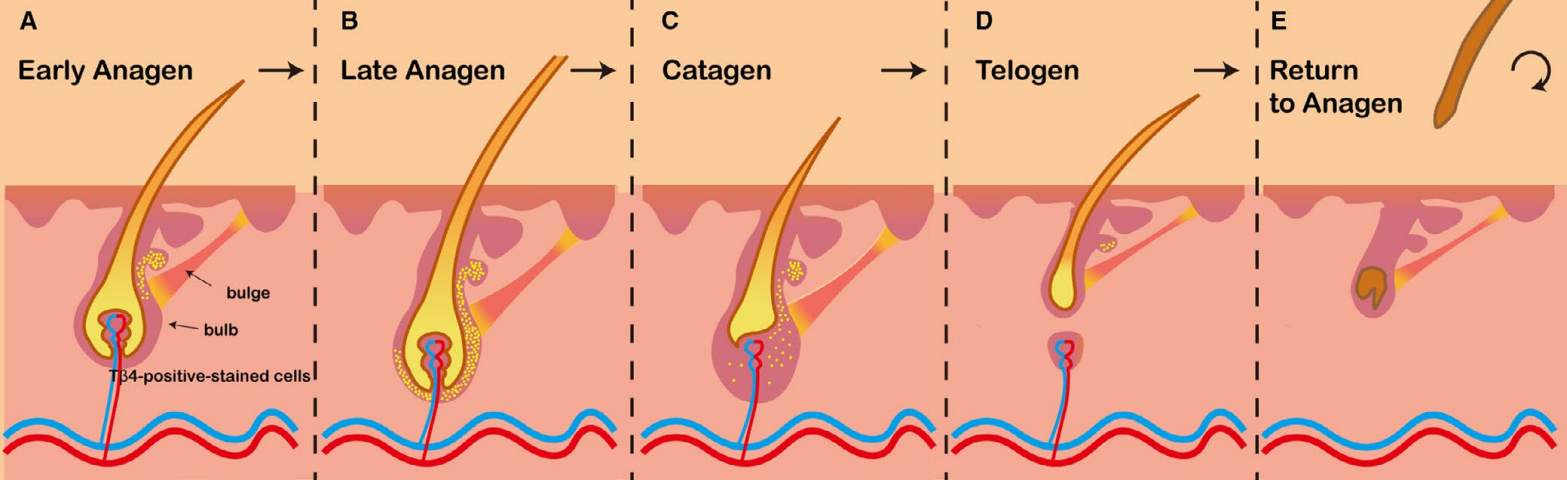
Tβ4 increases hair growth by the activation of hair follicle stem cells. (A) Hair follicle transition to early anagen phase was associated with an increased number of Tβ4‐expressing cells in the bulge region. Moreover, some Tβ4‐positive–stained cells were detected in the lower part of the follicle, between the bulge and bulb area. (B) At late anagen phase, a significant number of the cells located in the lower follicle expressed Tβ4. (C) At catagen phase, the number of Tβ4‐expressing cells should theoretically decrease. Philp et al showed that during this process, Tβ4‐positive cells expressed patterns similar to those of hair follicle stem cells (D) Low levels of Tβ4 were observed in follicles at the telogen phase. In these follicles, Tβ4 expression was confined to a small number of cells residing in the bulge region. (E) The roots of the hair fall off and new hair forms at the base of the hair follicle^34^

According to the literature, few studies have assessed the roles of endogenous Tβ4 in HF growth and development, and most studies on this topic have been published by a single group.^35^ The primary goal of this research group is to evaluate the roles of endogenous Tβ4 in the growth and development of HFs in mice and rats. In mice, they have focused on the roles of endogenous Tβ4 at different stages of the growth and development of mature HFs. During the telogen phase in mouse mature HFs, endogenous Tβ4 is expressed only in a small number of cells in the bulge region. When HFs in mice gradually enter the early stage of growth, cells in the bulge region of the HF exhibit significant increases in the number of Tβ4‐positive cells. At the same time, Tβ4‐positive cells are also present between the follicle bulge region and the bulb region. During the late anagen stage of HF growth, Tβ4 is expressed in a large number of cells in the HF bulb region, indicating that Tβ4‐positive cells are constantly migrating to the HF bulb region. Interestingly, this trend of metastasis is consistent with the trend of differentiation of HF stem cells. Moreover, HF stem cells also transfer from the bulge region to the hair bulb region to induce the formation of matrix cells and eventually differentiate into the hair shaft. This suggests that endogenous Tβ4 may be related to the differentiation of early HF stem cells. To further investigate this hypothesis, the Philp team isolated and cultured keratinized cells from the HF bulge region of rat vibrissa. These cells may represent HF stem cells, which express keratin 15 as a specific molecular marker. Philp found that Tβ4 expression could be detected in keratinocytes after 7‐10 days of culture. At the same time, the expression of keratin 15 decreased after exogenous Tβ4 treatment, indicating that endogenous Tβ4 activated the transformation of the HF cycle and affected the growth and development of HFs by promoting the migration and differentiation of HF stem cells and their progeny (Figure 3).

Endogenous Tβ4 can also indirectly promote the growth and development of HFs by stimulating angiogenesis. Angiogenesis plays key roles in some important physiological processes, such as wound healing and tumour growth, and is a key indicator of wound healing.^51^, ^52^, ^53^ Platelets and polymorphonuclear white blood cells at the site of injury have been reported to have higher levels of Tβ4 in different animal models because they are the first cells to enter the wound and release factors.^51^, ^54^ Malinda et al first found that Tβ4 could activate the migration of keratinocytes and promote the healing of skin wounds in a full‐layer skin injury model in rats.^55^ Further studies have shown that Tβ4 also promotes the differentiation of basement membrane cells around the damaged area into epidermal cells and induces the migration of these cells to the damaged area; additionally, Tβ4 also promotes the migration of keratinocytes from the injury edge to the injury centre and induces the redifferentiation of wound cells.^35^, ^55^, ^56^, ^57^ The key cells promoting the growth of vascular epithelial tissues are neutrophils and platelets, and the concentration of Tβ4 is highest in these cells,^51^ promoting the activation of inflammatory cells, expression of angiogenic factors, and differentiation and migration of vascular endothelial cells. Grant et al demonstrated that *Tβ4* mRNA expression in endothelial cells increased fivefold during vascular tubular formation.^48^ Furthermore, Cha et al showed that the activity of Tβ4‐induced angiogenesis may be related to the expression level of vascular endothelial growth factor (VEGF) and that the activity of Tβ4‐induced angiogenesis increases when the expression level of VEGF increases. In addition, hepatocellular growth factor, another angiogenesis‐promoting protein, is expressed in HFs and has been found to promote HF growth and development.^34^ Hepatocyte growth factor up‐regulates the expression of Tβ4 during angiogenesis, suggesting that hepatocyte growth factor may indirectly affect HF growth and development by enhancing the expression of Tβ4 or synergistically exerting biological effects with Tβ4.^58^, ^59^ Therefore, the cyclic circulation of the HF and the regulation of hair growth can be achieved indirectly through endogenous Tβ4‐dependent angiogenesis around the HF.^54^, ^60^, ^61^

In summary, endogenous Tβ4 is involved in HF growth and development by promoting the migration and differentiation of HF stem cells and their progeny. Additionally, endogenous Tβ4 can also indirectly affect the growth and development of HFs by promoting angiogenesis around the HF. However, few studies have evaluated the roles of endogenous Tβ4; thus, additional studies are required.

## 4 EFFECTS OF EXOGENOUS Tβ4 ON HF GROWTH AND DEVELOPMENT

The Philp team first identified the effects of exogenous Tβ4 on HF growth and development by treatment of the skin surface with Tβ4.^34^, ^35^ During their study of wound healing in the skin of rats, they unexpectedly observed that, at the histological level, the number of HFs at the wound margins increased after 7 days of topical treatment with Tβ4. In this study, the sides of healthy rats were shaved, and Tβ4 or control vehicle was applied to each side. After 7 days of treatment, the number of growth follicles in the skin area treated with Tβ4 increased to approximately twice that in the skin area treated with control vehicle. After 3 weeks of continuous treatment with Tβ4, the new hair persisted for more than 30 days. However, within 14 days of stopping treatment, the number of follicles dropped to the original level. In addition, researchers examined whether Tβ4 promoted hair growth in 8‐week‐old C57BL6 wild‐type mice and showed that mice treated with Tβ4 exhibited rapid hair regeneration. Histological examination also confirmed the function of Tβ4 in activating the transition of the HF cycle. However, although the application of exogenous Tβ4 on the body surface indicated that Tβ4 influenced the growth and development of HFs, the methods used in these studies were relatively simplistic. Owing to limitations of time and treatment, these studies did not fully elucidate how Tβ4 affects the growth and development of HFs.

With the establishment and improvement of cloning technology, the influence of exogenous Tβ4 on the growth and development of HFs was also investigated through the generation of a mouse model overexpressing Tβ4. In 2007, Philp et al^34^ generated transgenic mice overexpressing Tβ4 driven by the keratin 5 promoter. When the rate of hair regrowth was measured after shaving the hair of resting 8‐week‐old mice, hair in Tβ4‐overexpressing mice grew faster than that in wild‐type mice from the same litter. Such results were also verified in mouse models generated by Cha et al In 2010, Cha et al^62^ also generated transgenic mice that overexpressed Tβ4 driven by the keratin 5 promoter. They demonstrated that the overexpression of Tβ4 accelerated hair growth in mice by up‐regulation of laminin 5. These results were consistent with another two studies. First, Chuang et al^63^ confirmed that laminin 5 is highly expressed in HFs. Additionally, Brakebusch et al^64^ demonstrated that the integrity of HFs depended on expression of integrin β1, the major receptor component of laminin 5, in keratinocytes. In 2015, Liu et al^38^ also generated mice overexpressing Tβ4. However, they used keratin 14 as the promoter to drive Tβ4 expression. The results showed that the hair of mice overexpressing Tβ4 was longer and thicker than that of wild‐type mice. Moreover, they confirmed the roles of Tβ4 by generating Tβ4‐knockout mice using the most advanced TALEN technology at the time. The results showed that after shaving the backs of mice, the skin of Tβ4‐knockout mice became darker, and the hair regeneration rate was significantly lower than that in wild‐type mice. In summary, in the last decade of research, different teams have shown that exogenous Tβ4 promotes the growth and development of HFs in mice and increases the rate of hair growth.

**FIGURE 4:**
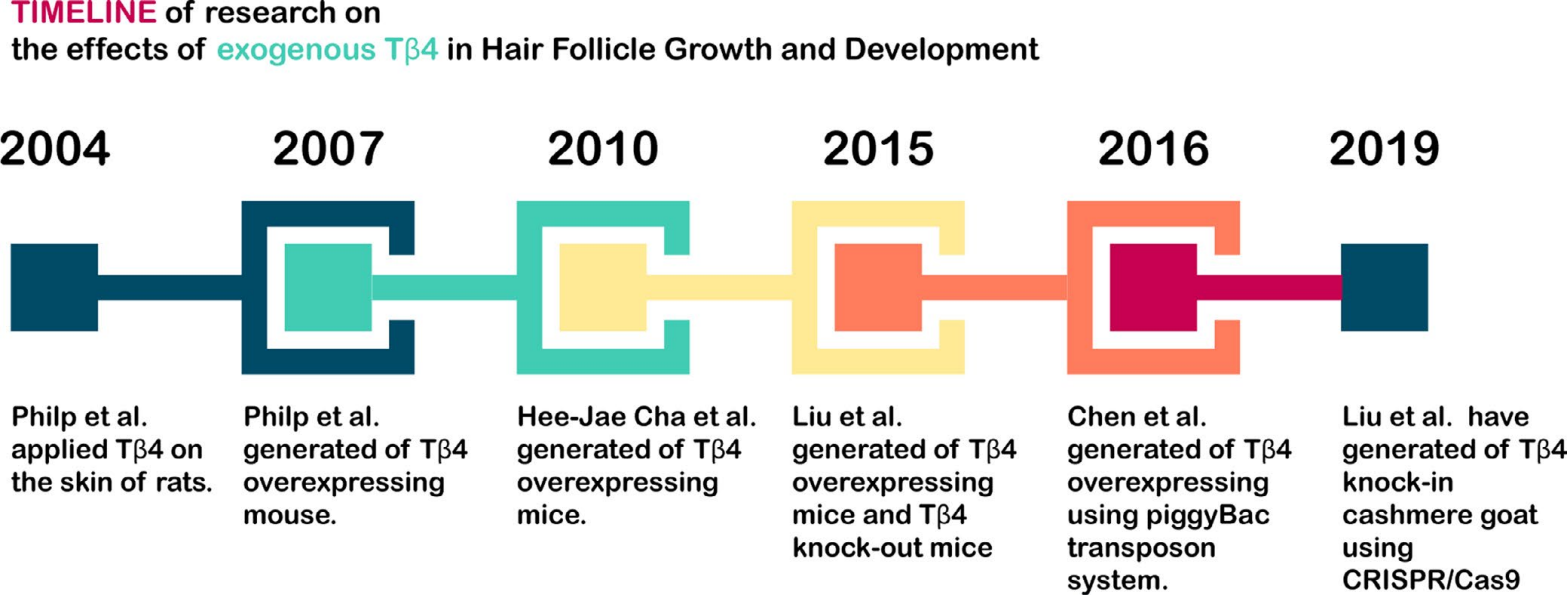
Timeline of research on the effects of exogenous Tβ4 in hair follicle growth and development

Increased cashmere yield and improved quality are some goals of Alpas cashmere goat breeding. Cashmere is produced from secondary HFs of cashmere goats. Based on the above study of exogenous Tβ4 in mouse models, researchers began to explore the roles of exogenous Tβ4 in the growth and development of secondary HFs in cashmere goats. In 2016, Chen et al^65^ used the piggyBac transposon system to successfully generate exogenous Tβ4‐expressing cashmere goats in which gene expression was driven by keratin‐associated protein 6.1 (KAP6.1) and specifically expressed in HFs. Dermal histology revealed that transgenic cashmere goats with Tβ4 overexpression had a higher ratio of secondary to primary follicles than wild‐type cashmere goats, suggesting that the overexpression of Tβ4 increased the number of secondary follicles in cashmere goats. Therefore, the specific overexpression of Tβ4 in the skin follicles of cashmere goats is a feasible strategy for improving cashmere yields. However, the random integration of exogenous genes may lead to silencing of gene expression, which may not accurately reflect the influence of exogenous genes on phenotype. The development of gene‐editing technology, particularly CRISPR‐Cas9 technology, has provided technical support for solving this problem. In 2019, Liu et al^36^ used CRISPR‐Cas9 technology to generate cashmere goats integrated with exogenous Tβ4 at the C‐C chemokine receptor type 5 (CCR5) safety site. They also added a KAP6.1 promoter prior to the addition of exogenous Tβ4, ensuring accurate expression in skin follicles. The results showed that the cashmere yield of fixed‐point integrated exogenous Tβ4 cashmere goats was significantly higher than that of wild‐type cashmere goats. Overall, these findings showed that exogenous Tβ4 not only enhances the growth rate of hair but also increases the amount of hair produced. Thus, the influence of exogenous Tβ4 on the growth and development of HFs may be multifaceted (Figure 4).

## 5 POSSIBLE SIGNALLING PATHWAYS OF Tβ4 IN HF GROWTH AND DEVELOPMENT

Tβ4 regulates the growth and development of HFs by promoting the synthesis and secretion of matrix metalloproteinase (MMP)‐2. When Philp et al^34^, ^35^ added exogenous Tβ4 to HF stem cells, they found that MMP‐2 showed a concentration‐dependent increase in expression. Because MMP‐9 expression did not change following treatment with Tβ4, Tβ4 is thought to mainly promote the secretion of MMP‐2. MMP‐2, a member of the MMP protease family, is a 72‐kDa protease with Zn^2+^ as cofactor secreted by matrix cells. MMP‐2 is expressed in most normal tissues and is able to degrade denatured collagen (types I, III, IV, V, VII, X and XI collagen), fibronectin, vitronectin, laminin, versican and several non‐matrix substrates.^66^ The degradation function of MMP‐2 can reshape the extracellular matrix, thereby separating the epidermis of the HF (such as the outer root sheath) from the basement membrane of the matrix layer (such as the dermal sheath and DP).^67^ This process is essential for signalling between epithelial and matrix components in the HF and for the extension and invasion of the HF into the subcutaneous tissue in the lower part during the growth phase. Moreover, MMP‐2 can be remodelled by degradation of laminin 5, the key component of the basement membrane. Cha et al^62^, ^67^ also found that the overexpression of Tβ4 accelerates hair growth in mice by up‐regulating laminin 5. Thus, Tβ4 can remodel the basement membrane of HFs by promoting the secretion of MMP‐2 via up‐regulation of laminin 5.

Tβ4 can also promote angiogenesis and affect the growth and development of HFs through VEGF. Liu et al^38^ found that compared with wild‐type mice, the expression of VEGF in the skin of Tβ4‐overexpressing mice was increased. Notably, the increased expression of VEGF is induced by up‐regulation of hypoxia‐inducible factor 1 by Tβ4.^54^ Moreover, the proliferation, migration, differentiation and angiogenesis of endothelial progenitor cells are realized by the activation of the phosphatidylinositol 3‐kinase (PI3K)/AKT/endothelial nitric oxide synthase signalling pathway by Tβ4.^61^ Other studies have shown that the expression of VEGF is regulated by basic fibroblast growth factor (bFGF).^68^ At the early stage of wound healing, Tβ4 maintains high expression of VEGF and bFGF, activates the proliferation and differentiation of fibroblasts and the regeneration and migration of epithelial cells, and promotes angiogenesis.^55^, ^69^, ^70^, ^71^, ^72^, ^73^ Taken together, the above studies have shown that Tβ4 may affect the growth and development of HFs via VEGF.

The Wnt signalling pathway is another possible target of Tβ4 and is involved in regulating the growth and development of HFs.^74^, ^75^ Indeed, the Wnt signalling pathway is the main signalling pathway controlling HF growth; β‐catenin and lymphoid enhancer‐binding factor 1 (LEF‐1) are key molecules in this pathway. In the nucleus, β‐catenin directly interacts with LEF‐1, activates the keratin gene, promotes hair growth and regulates the HF cycle.^76^, ^77^, ^78^, ^79^ In addition, after Wnt signalling is activated, β‐catenin can also bind to the TCF/LEF complex to form the β‐catenin/TCF/LEF complex. In a study by Liu et al,^37^, ^38^ compared with wild‐type mice, the expression levels of β‐catenin and LEF‐1 were increased in mice overexpressing Tβ4. This result suggests that Tβ4 can affect the Wnt signalling pathway. Interestingly, VEGF is a target protein of the β‐catenin/TCF/LEF complex and has been reported to directly affect the secretion of histone proteins, including MMPs, thereby linking VEGF with MMP‐2. In addition, Liu et al also showed that the p38 mitogen–activated protein kinase, extracellular signal–regulated kinase 1/2 and PI3K/AKT pathways were also activated in Tβ4‐overexpressing mice.

**FIGURE 5:**
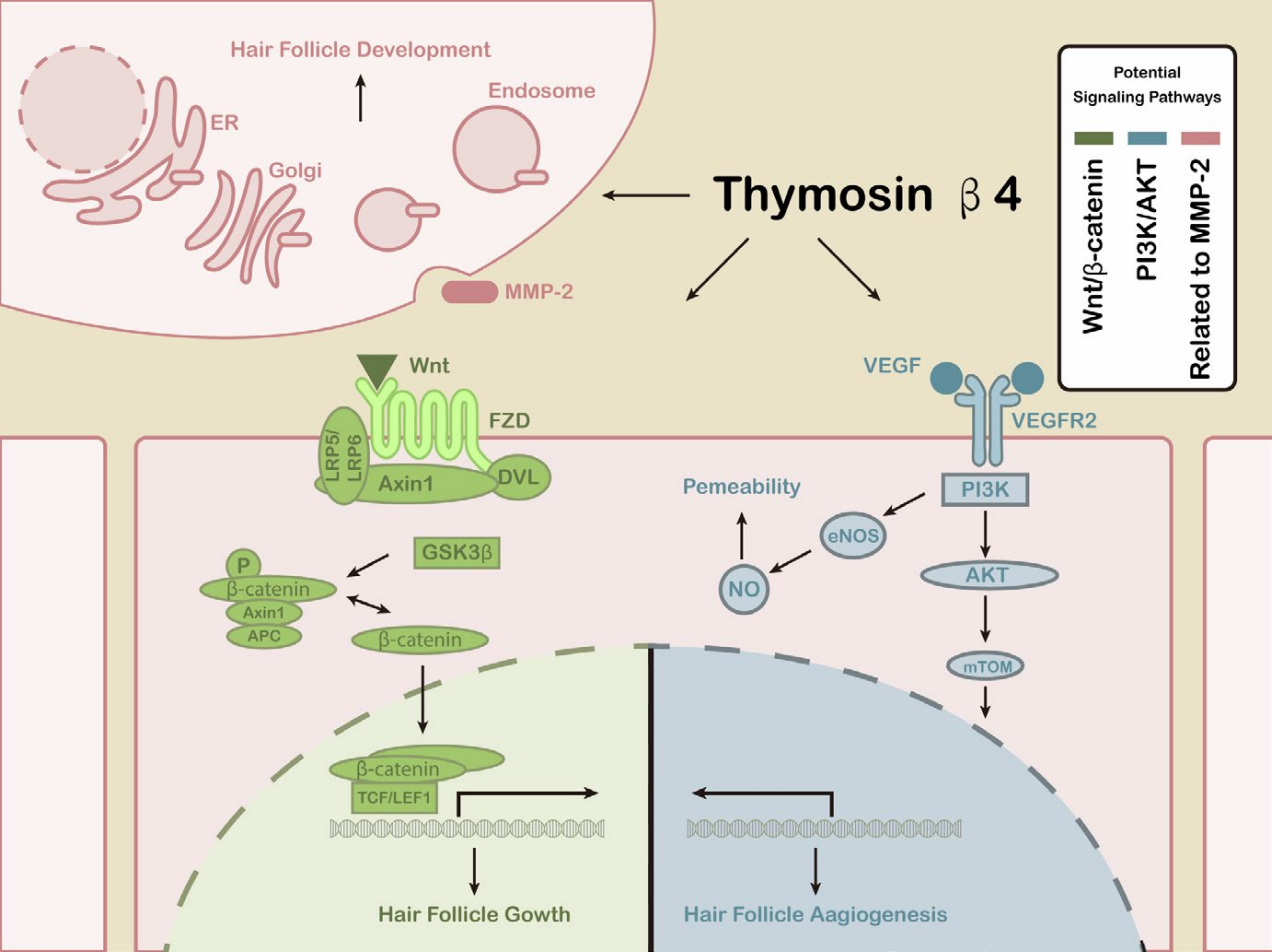
Potential signalling pathways of Tβ4 in hair follicle growth and development. There are three main signalling pathways: Wnt/β‐catenin, phosphatidylinositol 3‐kinase (PI3K)/AKT and matrix metalloproteinase (MMP)‐2. VEGF: vascular endothelial growth factor; VEGFR: VEGF receptor; eNOS: endothelial nitric oxide synthase; NO: nitric oxide; ER: endoplasmic reticulum; FZD: frizzled; DVL: dishevelled; APC: adenomatous polyposis coli

Taken together, these studies suggested that Tβ4 may influence the growth and development of HFs through different signalling pathways (Figure 5). These signalling pathways are not independent of each other, and various studies have shown that they are interconnected. However, further studies are still needed to confirm this relationship in the growth and development of HFs.

## 6 CONCLUSIONS

The growth and development of HFs involve many molecules and cells. Therefore, the search for key signalling molecules that control these processes has become a major focus of research. In recent years, the study of Tβ4 in the growth and development of HFs has gradually become a research hotspot in this field, not only because of its involvement in cell differentiation, angiogenesis, wound healing and other biological processes but also because of its potential clinical and production application value. In terms of HF growth and development, endogenous Tβ4 can activate the transformation of the HF cycle in mice and affect the growth and development of HFs by promoting the migration and differentiation of HF stem cells and their progeny. Exogenous Tβ4 increases the rate of hair growth in mice and promotes cashmere production by increasing the number of secondary follicles in cashmere goats. Moreover, Tβ4 can affect the growth and development of HFs by promoting the synthesis and secretion of MMP‐2, angiogenesis‐inducing effects of VEGF and activation of the Wnt signalling pathway.

However, most studies have been limited to the analysis of Tβ4 expression in HFs and the generation of animal models modified by Tβ4. The mechanisms through which Tβ4 affects the growth and development of HFs are still unclear. With the continuous development of systems biology, genomics, bioinformatics and HF biology, our understanding of the roles of Tβ4 in HF growth and development will improve, providing insights into the potential applications of Tβ4 in human clinical and production fields.

## CONFLICTS OF INTEREST

The authors declare no conflicts of interest.

## AUTHOR CONTRIBUTION

**bai dai:** Formal analysis (equal); Investigation (equal). **rina sha:** Data curation (equal); Resources (equal); Software (equal); Writing‐original draft (equal). **jianlong yuan:** Investigation (equal); Methodology (equal); Project administration (equal); Writing‐review & editing (equal). **dongjun liu:** Conceptualization (equal); Supervision (equal); Validation (equal).
